# Chronic Occupational Stress and Immune–Inflammatory Dysregulation: Mechanisms, Clinical Consequences, Biomarkers and Implications for Occupational Medicine

**DOI:** 10.3390/diagnostics16193097

**Published:** 2026-09-24

**Authors:** Ancuța-Ramona Boicea Camen, Mihail Virgil Boldeanu, Daniel Cosmin Caragea, Dan Nicolae Florescu, Lidia Boldeanu, Isabela Siloși, Mohamed-Zakaria Assani

**Affiliations:** 1 Department of Occupational Medicine, University of Medicine and Pharmacy of Craiova, 200349 Craiova, Romania; ancuta.boicea@umfcv.ro; 2 Department of Immunology, Faculty of Medicine, University of Medicine and Pharmacy of Craiova, 200349 Craiova, Romania; mihail.boldeanu@umfcv.ro (M.V.B.); isabela.silosi@umfcv.ro (I.S.); mohamed.assani@umfcv.ro (M.-Z.A.); 3 Department of Nephrology, Faculty of Medicine, University of Medicine and Pharmacy of Craiova, 200349 Craiova, Romania; daniel.caragea@umfcv.ro; 4 Department of Gastroenterology, Faculty of Medicine, University of Medicine and Pharmacy of Craiova, 200349 Craiova, Romania; 5 Research Center of Gastroenterology and Hepatology, University of Medicine and Pharmacy of Craiova, 200638 Craiova, Romania; 6 Department of Microbiology, Faculty of Medicine, University of Medicine and Pharmacy of Craiova, 200349 Craiova, Romania; 7 Doctoral School, University of Medicine and Pharmacy of Craiova, 200349 Craiova, Romania

**Keywords:** occupational stress, chronic stress, inflammation, psychoneuroimmunology, inflammatory biomarkers, occupational medicine

## Abstract

Chronic occupational stress is associated with adverse cardiovascular, metabolic, and mental-health outcomes, but the biological evidence linking workplace exposures to immune–inflammatory dysregulation varies in directness and clinical relevance. This narrative review distinguishes findings demonstrated directly in occupational populations from mechanisms extrapolated from general psychological-stress, experimental, and preclinical models, and evaluates the potential utility of candidate biomarkers in occupational health. Prospective occupational evidence supports a weak and heterogeneous association between adverse psychosocial working conditions and inflammatory activity, with the clearest signal reported for IL-6. Mechanistic studies implicate hypothalamic–pituitary–adrenal axis dysregulation, altered sympathetic signaling, reduced glucocorticoid sensitivity, NF-κB-related transcription, cytokine responses, immune-cell alterations, oxidative stress, and stress-responsive gene regulation; however, much of this evidence derives from non-occupational models and primarily supports biological plausibility. Occupational epidemiological evidence is strongest for cardiovascular and selected metabolic outcomes, while associations with depressive symptoms and psychological distress are recurrent but remain limited by concerns regarding causal inference. Evidence linking occupational stress specifically to autoimmune and skeletal outcomes remains comparatively indirect. C-reactive protein, IL-6, salivary cortisol, and hair cortisol remain research-oriented measures because none has demonstrated sufficient occupational-stress specificity, validated diagnostic accuracy, clinically actionable thresholds, or incremental predictive value beyond established psychosocial and clinical assessment. Routine biomarker-based diagnosis or surveillance is therefore not currently supported. Future research should prioritize repeated assessment of occupational exposures and biomarkers, temporally appropriate mediation analyses, independent replication, and external validation before biological monitoring can inform routine occupational-health practice.

## 1. Prevalence of Chronic Occupational Stress as a Public Health Issue

Psychosocial stress at work represents an important occupational and public health problem because prolonged exposure to adverse psychosocial working conditions has been associated with increased risks of cardiovascular, metabolic, psychological, and immune-related outcomes [1,2].

Occupational psychosocial stress encompasses several distinct exposures, including high job demands, low job control, job strain, effort–reward imbalance, job dissatisfaction, burnout, organizational downsizing, and other unfavorable characteristics of the working environment [2,3]. The prevalence of occupational stress cannot be represented reliably by a single global percentage because available studies use different definitions of stress, different psychometric instruments, different thresholds for exposure, and markedly different occupational populations [2,3].

The public health burden of psychosocial occupational exposures is supported by European population-level estimates linking job strain, effort–reward imbalance, job insecurity, long working hours, and workplace bullying with substantial burdens of coronary heart disease, stroke, atrial fibrillation, peripheral artery disease, and depression [4]. In an analysis of 28 European countries, psychosocial occupational exposures were estimated to account for 173,629 disability-adjusted life years, DALYs, attributable to coronary heart disease among men and 39,238 DALYs among women in 2015 [4]. The estimated burden attributable to the same psychosocial occupational exposures was even higher for depression, reaching 528,549 DALYs in men and 344,151 DALYs in women [4].

Prospective epidemiological evidence also indicates that workplace stress is associated with subsequent cardiovascular morbidity rather than exclusively with subjective psychological distress [1].

A review including more than 600,000 participants from 27 cohort studies conducted in Europe, the United States, and Japan found that job strain and long working hours were associated with a moderately elevated risk of incident coronary heart disease and stroke [1]. The excess cardiovascular risk reported in exposed workers was approximately 10% to 40% compared with individuals who were not exposed to these occupational stressors [1]. The same review also identified an association between work-related stress and type 2 diabetes, whereas comparable associations were not demonstrated for common cancers or chronic obstructive pulmonary disease, suggesting some degree of disease specificity [1].

Chronic psychological stress can influence immune function through interactions between neuroendocrine pathways and immune cells [2]. Occupational psychosocial stress has been associated with alterations in several immune parameters, including reduced natural killer, NK, cell activity, changes in NK-cell and T-cell subsets, alterations in the CD4+/CD8+ ratio, and increased inflammatory markers [2]. These observations indicate that chronic occupational stress may affect both innate and adaptive components of the immune response [2,3].

Low-grade systemic inflammation is clinically relevant because it has been associated with an increased risk of cardiovascular disease, diabetes, depression, and other chronic disorders [5].

Longitudinal occupational research has therefore investigated inflammatory biomarkers such as C-reactive protein, CRP, and interleukin-6, IL-6, as possible biological intermediates between adverse psychosocial working conditions and chronic disease [5].

In the PROspective Quebec Study on Work and Health, psychosocial occupational exposures were assessed using established models including the Demand–Control–Support model and the Effort–Reward Imbalance model, while CRP and IL-6 were measured using standardized protocols [5].

Among men, exposure to iso-strain was associated with an increased prevalence of a high inflammatory index, with a prevalence ratio of 1.42 and a 95% confidence interval of 1.06 to 1.90 [5]. Among men younger than 65 years, the corresponding prevalence ratio reached 2.00, with a 95% confidence interval of 1.37 to 2.92 [5]. Associations with inflammatory biomarkers were also observed in some groups exposed to effort–reward imbalance, low occupational reward, or moderate social support at work [5]. The investigators concluded that psychosocial stressors at work may contribute to low-grade inflammation, while emphasizing that further studies are required to confirm these relationships and clarify sex-specific differences [5].

Psychological stress has also been associated with metabolic syndrome in quantitative evidence synthesis [6]. A systematic review and meta-analysis reported that adults exposed to high psychological stress had 45% higher adjusted odds of metabolic syndrome than adults in low-stress groups, with a pooled odds ratio of 1.45 and a 95% confidence interval of 1.21 to 1.74 [6]. In analyses stratified according to the source of stress, occupational stress showed the strongest association with metabolic syndrome, with an odds ratio of 1.69 and a 95% confidence interval of 1.18 to 2.42 [6]. However, the occupational-stress analysis was largely based on cross-sectional evidence, and the authors explicitly identified the scarcity of longitudinal studies as an important limitation [6].

Mechanistically, chronic stress may promote persistent inflammation through altered neuroendocrine regulation, including reduced glucocorticoid sensitivity and increased pro-inflammatory transcriptional activity [7,8,9].

Collectively, available evidence supports a biological framework in which prolonged psychosocial occupational stress can alter neuroendocrine–immune regulation, modify immune-cell function, impair glucocorticoid control of inflammation, and promote persistent inflammatory signaling [2,7,8]. These mechanisms provide a biologically plausible connection between chronic occupational stress and the increased cardiovascular and metabolic risks observed in epidemiological studies [1,6].

Previous syntheses have examined psychosocial job stress in relation to immune function, inflammatory biomarkers, cardiovascular risk, and selected neuroendocrine markers, but these evidence bases include markedly different exposure contexts and study designs. A central unresolved question is therefore not whether psychological stress can influence immune and neuroendocrine biology in principle, but which of these mechanisms have been demonstrated directly in occupational populations and whether any resulting biological measurements provide clinically useful information beyond established psychosocial and conventional clinical assessment. This distinction between biological plausibility, occupational evidence, and clinical validity is particularly relevant to the diagnostic and monitoring perspective of the present review.

This review aims to assess the directness and clinical relevance of evidence linking chronic occupational stress with neuroendocrine and immune–inflammatory dysregulation. Evidence is distinguished among: (i) findings demonstrated directly in occupational populations; (ii) mechanistic findings derived from non-occupational human psychological-stress models; and (iii) preclinical or experimental evidence supporting biological plausibility. Within this framework, the review evaluates cardiovascular, metabolic, immune-mediated, and mental-health outcomes and critically examines candidate biomarkers according to their potential use for exposure identification, clinical diagnosis, risk prediction, and longitudinal monitoring. Particular attention is given to the evidence that would still be required before biological measures could be incorporated into routine occupational-health decision making.

## 2. Approach to Literature Search and Review Process

### 2.1. Literature Search and Evidence Synthesis

This narrative review was developed through a structured literature search focused on the neuroendocrine, immunological, inflammatory, occupational, and clinical consequences of chronic work-related stress. The literature search was conducted using PubMed with emphasis on peer-reviewed publications relevant to psychoneuroimmunology and occupational medicine. No lower publication-date restriction was applied, and the literature search covered records available from database inception to 11 September 2026. Only peer-reviewed publications available were considered. Because this was a narrative rather than a systematic review, study identification and selection were not conducted according to a PRISMA-based systematic-review protocol.

The search strategy was organized according to the major conceptual domains addressed in the review. Search terms were used individually and in combinations and included: “occupational stress”, “work stress”, “job strain”, “psychosocial stress”, “chronic stress”, “workplace stress”, “effort-reward imbalance”, “job demand-control”, “burnout”, “workplace bullying”, “mobbing”, “shift work”, “night work”, “HPA axis”, “cortisol”, “glucocorticoid resistance”, “sympathetic nervous system”, “catecholamines”, “norepinephrine”, “inflammation”, “NF-kappa B”, “IL-6”, “TNF-alpha”, “IL-1 beta”, “C-reactive protein”, “immune cells”, “natural killer cells”, “T lymphocytes”, “monocytes”, “oxidative stress”, “inflammaging”, “DNA methylation”, “epigenetics”, “conserved transcriptional response to adversity”, “cardiovascular disease”, “atherosclerosis”, “metabolic syndrome”, “insulin resistance”, “type 2 diabetes”, “autoimmune disease”, “depression”, “anxiety”, “inflammatory biomarkers”, “salivary cortisol”, “hair cortisol”, “occupational health surveillance”, “mindfulness”, and “physical exercise”. A representative executable PubMed search strategy was: ((“occupational stress”[Title/Abstract] OR “work stress”[Title/Abstract] OR “job strain”[Title/Abstract] OR “workplace stress”[Title/Abstract] OR “psychosocial work stress”[Title/Abstract] OR “effort-reward imbalance”[Title/Abstract]) AND (inflamm*[Title/Abstract] OR cytokine*[Title/Abstract] OR “C-reactive protein”[Title/Abstract] OR CRP[Title/Abstract] OR “interleukin-6”[Title/Abstract] OR “IL-6”[Title/Abstract] OR “TNF-alpha”[Title/Abstract] OR “IL-1 beta”[Title/Abstract] OR cortisol[Title/Abstract] OR “HPA axis”[Title/Abstract] OR “glucocorticoid resistance”[Title/Abstract] OR immun*[Title/Abstract])). Additional targeted searches combined occupational-stress terms with individual exposures, mechanisms, biomarkers, and clinical outcomes addressed in the review.

Searches were refined iteratively as specific mechanisms and clinical outcomes were identified during manuscript development. Additional publications were identified by examining reference lists and related articles from relevant systematic reviews, meta-analyses, longitudinal cohort studies, and mechanistic studies.

Priority was given to systematic reviews and meta-analyses when evaluating the overall strength and consistency of associations between occupational stress and health outcomes. Large prospective cohort studies and individual-participant-data meta-analyses were preferentially used for cardiovascular, metabolic, and psychiatric outcomes because these designs provide stronger evidence regarding temporal relationships than cross-sectional studies. Original human studies were prioritized when discussing specific biological mechanisms, including glucocorticoid resistance, inflammatory cytokine responses, NF-κB-related transcriptional activity, immune-cell function, cortisol dynamics, and stress-related gene-expression patterns.

Cross-sectional occupational studies were included when longitudinal evidence was limited or when they provided relevant information regarding specific occupational populations, including healthcare workers, law-enforcement personnel, shift workers, and other high-demand occupations. Findings derived from cross-sectional studies were interpreted as associations and were not considered evidence of causality.

Experimental and mechanistic studies of psychological stress were included when they provided biologically relevant evidence for pathways that have not yet been sufficiently characterized in occupational populations. In such cases, evidence obtained from general chronic-stress models was distinguished from evidence directly demonstrated in workers, and mechanisms identified outside occupational settings were not assumed to represent occupational-specific effects.

Attention was given to studies assessing established occupational stress models, including the Job Demand–Control model and the Effort–Reward Imbalance model, as well as studies evaluating specific psychosocial exposures such as job insecurity, long working hours, night and shift work, workplace bullying, and occupational burnout.

For the evaluation of potential biomarkers, studies examining C-reactive protein, IL-6, other inflammatory mediators, salivary cortisol, and hair cortisol were reviewed with particular attention to biological specificity, temporal variability, sampling methodology, confounding factors, and evidence for application in occupational populations. Studies evaluating biomarkers solely as research outcomes were distinguished from those investigating their potential use in occupational health surveillance.

Intervention studies were considered when they provided relevant evidence regarding changes in psychological or biological stress-related outcomes. Because intervention evidence was not a primary focus of the present review, these studies were interpreted mainly in relation to biomarker responsiveness and the potential implications for occupational-health monitoring.

Only scientific claims that could be traced to identifiable peer-reviewed publications were retained in the review. Bibliographic information and scientific claims were verified against the original peer-reviewed publication before inclusion in the manuscript. Particular care was taken to avoid extrapolating findings from a specific profession, biomarker, or stress model to occupational stress in general.

Evidence was synthesized narratively because of substantial heterogeneity in occupational stress definitions, exposure assessment instruments, study populations, biological sampling procedures, biomarker assays, follow-up periods, and reported clinical outcomes. No quantitative meta-analysis was performed as part of the present review.

The interpretation of evidence followed a hierarchical approach in which meta-analyses, systematic reviews, prospective cohort studies, randomized or controlled intervention studies, and mechanistic human investigations were generally given greater weight than isolated cross-sectional studies. However, study design, sample size, methodological limitations, population characteristics, and consistency with the broader literature were considered when evaluating individual findings.

The review specifically distinguished biological plausibility from established clinical or occupational-health utility. Accordingly, biomarkers and mechanisms supported by experimental or observational research were not considered validated diagnostic or screening tools unless sufficient occupational evidence was available. This distinction was particularly important for glucocorticoid resistance, inflammatory cytokines, epigenetic changes, the conserved transcriptional response to adversity, and hair cortisol, for which mechanistic relevance currently exceeds their validated application in routine occupational medicine.

The final synthesis was organized around the sequential relationship between occupational psychosocial exposures, neuroendocrine stress responses, immune–inflammatory dysregulation, clinical consequences, candidate biomarkers, and preventive strategies. This approach was selected to integrate evidence from immunology and occupational medicine while maintaining a clear distinction between mechanistic findings, epidemiological associations, and potential clinical applications.

### 2.2. Framework for Evidence Interpretation

To improve transparency in the interpretation of heterogeneous evidence, the studies included in this review were considered according to both methodological quality and directness to occupational exposure. Evidence derived from prospective or longitudinal occupational studies was given the greatest weight when evaluating associations between workplace stressors and biological or clinical outcomes. Cross-sectional occupational studies were considered supportive but insufficient for causal inference. Human studies of chronic psychological stress conducted outside occupational settings were used primarily to clarify biological mechanisms, whereas animal and experimental models were treated as indirect mechanistic evidence. These categories were not considered equivalent, and mechanistic plausibility was not assumed to demonstrate occupational specificity or clinical validity. Special focus was also placed on factors such as sample size, how exposure was defined, biomarker measurement methods, adjustments for confounding variables, the sequence of events, risk of bias, and the consistency across different independent cohorts. Where multiple reports originated from the same cohort, these were interpreted as related analyses rather than independent replication.

Throughout the synthesis, major findings are therefore described as direct occupational evidence, indirect human mechanistic evidence, or preclinical/mechanistic evidence where appropriate. Conclusions regarding biomarkers and clinical applications were based not only on study design but also on directness, methodological limitations, consistency, and evidence of clinical validity.

Variables such as sleep, adiposity, smoking, physical activity, and metabolic abnormalities were interpreted according to the role assigned in the original study, as potential confounders, mediators, or parallel pathways; these roles were not assumed to be interchangeable.

### 2.3. Use of Generative Artificial Intelligence

ChatGPT (GPT-5.5, OpenAI) was used as an assisting tool during manuscript preparation to support the organization of the review, structuring of individual sections, synthesis and presentation of the literature, and language refinement. The tool was not used as an autonomous source of scientific evidence. References and scientific claims were independently reviewed and verified by the authors using the original publications.

Generative AI (Claude Sonnet 5, Anthropic) was used during manuscript preparation to assist with (i) synthesizing and organizing relevant literature during the background research phase, (ii) editing and refining the language of the manuscript, and (iii) verifying and formatting the bibliographic references according to the journal’s citation style.

All AI-assisted text was critically reviewed and edited by the authors, who retain full responsibility for the scientific content and conclusions of the manuscript.

## 3. Neuroendocrine–Immune Axis in Chronic Stress

### 3.1. Hypothalamic–Pituitary–Adrenal Axis and Cortisol

The hypothalamic–pituitary–adrenal, HPA, axis is one of the principal neuroendocrine systems activated during the physiological response to psychological stress [10]. Stress-related activation of the HPA axis leads to increased secretion of glucocorticoids, predominantly cortisol in humans, which subsequently influences metabolic, cardiovascular, neural, and immune functions [10].

At the hypothalamic level, stress stimulates the release of corticotropin-releasing hormone, CRH, which promotes secretion of adrenocorticotropic hormone, ACTH, from the anterior pituitary gland [11]. ACTH subsequently stimulates the adrenal cortex to produce and release cortisol into the circulation [11]. Circulating cortisol participates in negative feedback regulation of the HPA axis by inhibiting further CRH and ACTH production, thereby limiting excessive activation of the stress response [11].

Cortisol secretion is normally characterized by circadian and pulsatile patterns, and preservation of these temporal dynamics contributes to physiological immune regulation and systemic homeostasis [11,12].

Glucocorticoids exert their cellular effects primarily through the glucocorticoid receptor, GR, a ligand-dependent transcription factor expressed in a broad range of tissues and immune cells [13].

Binding of glucocorticoids to GR alters the transcription of a large number of genes involved in metabolism, cellular homeostasis, immunity, and inflammation [12]. Glucocorticoid-responsive genes include numerous genes involved in immune and inflammatory responses [12].

One of the major physiological effects of glucocorticoid signaling is suppression of excessive inflammatory activity [10]. Activated glucocorticoid receptors inhibit inflammatory transcriptional pathways, including NF-κB signaling, thereby reducing transcription of several pro-inflammatory genes [10]. Through these mechanisms, cortisol can suppress production of pro-inflammatory cytokines including IL-1β, IL-6, and TNF-α [11].

Cortisol also modulates adaptive immunity by limiting lymphocyte proliferation and can reduce leukocyte migration toward inflammatory tissues [11]. The HPA axis and immune system communicate bidirectionally rather than functioning as independent systems [11]. Pro-inflammatory cytokines can stimulate components of the HPA axis, while glucocorticoids produced downstream provide negative feedback that restrains the inflammatory response [11]. This reciprocal regulation is particularly important during acute stress, when temporary activation of inflammatory and neuroendocrine responses can be followed by restoration of homeostasis [10].

Under chronic stress conditions, however, prolonged or repeated HPA-axis activation can lead to dysregulation of cortisol signaling rather than a simple sustained increase in cortisol concentration [11,14].

Chronic stress may therefore be associated with altered cortisol secretion patterns, impaired HPA-axis feedback, and reduced cellular responsiveness to glucocorticoids [11]. These alterations are important because circulating cortisol levels alone may not accurately reflect the biological effectiveness of glucocorticoid signaling within immune cells [15].

### 3.2. Sympathetic Nervous System and Catecholamines

The sympathetic nervous system, SNS, represents a second major pathway linking psychological stress with immune regulation [16]. Stress increases sympathetic activity and noradrenergic signaling, resulting in the release of norepinephrine from sympathetic nerve terminals and catecholamines from the adrenal medulla [10]. Epinephrine and norepinephrine can regulate immune function both systemically and locally [16]. Sympathetic nerve fibers innervate primary and secondary lymphoid tissues, providing a direct anatomical pathway through which neural signals can influence immune-cell behavior [16].

Immune cells express adrenergic receptors that allow them to respond directly to catecholamines released during sympathetic activation [16]. The β2-adrenergic receptor is particularly important in the regulation of immune-cell responses to catecholamines [16]. Adrenergic signaling can influence leukocyte migration, differentiation, proliferation, antibody production, and cytokine secretion, although the direction and magnitude of the effect depend on the immune-cell population, receptor subtype, ligand concentration, and inflammatory context [16].

Norepinephrine signaling and glucocorticoid signaling can exert opposing regulatory effects on inflammatory transcriptional pathways [10]. Noradrenergic signaling can increase the transcriptional activity of NF-κB, whereas glucocorticoid receptor signaling inhibits NF-κB activity through several molecular mechanisms [10]. Under physiological conditions, the balance between these pathways contributes to coordinated regulation of inflammatory responses during stress [10]. Chronic psychological stress can disturb this balance through persistent noradrenergic signaling and simultaneous reduction in the anti-inflammatory effectiveness of glucocorticoid signaling [10].

A systematic review including mouse, non-human primate, and human studies found that chronic stress was consistently associated with alterations in β-adrenergic signaling together with increased peripheral pro-inflammatory biomarkers [15]. The same systematic review included 15 mouse studies, 7 non-human primate studies, and 19 human studies evaluating glucocorticoid receptor and β-adrenergic receptor signaling during chronic stress [15].

Across these studies, chronic stress was associated with reduced cellular glucocorticoid sensitivity, alterations in intracellular β-adrenergic signaling, and increased pro-inflammatory biomarkers in peripheral blood [15]. Evidence reviewed in that analysis also suggested that stress-related alterations in glucocorticoid and adrenergic signaling may be particularly relevant to myeloid progenitor cells [15]. Stress-related neuroendocrine signaling may promote the release of myeloid cells that are intrinsically less sensitive to glucocorticoid-mediated suppression and show altered responsiveness to catecholamines [15].

The relationship between sympathetic activity and inflammation is nevertheless complex and cannot be described as uniformly pro-inflammatory [17]. Experimental and clinical evidence indicates that sympathetic influences can differ according to disease stage, receptor subtype, anatomical compartment, and duration of inflammatory exposure [17]. In chronic inflammatory disease models, sympathetic effects may be predominantly pro-inflammatory during early phases but may acquire anti-inflammatory characteristics after disease onset [17]. This context-dependent effect is important when interpreting catecholamines as biomarkers or mediators of occupational stress, because increased sympathetic activity does not translate into an identical immune response in every tissue or individual [17].

### 3.3. Glucocorticoid Resistance as a Mechanism of Persistent Inflammation

One of the central mechanisms proposed to explain chronic stress-associated inflammation is glucocorticoid resistance, also referred to as reduced glucocorticoid sensitivity [7,9,18]. Glucocorticoid resistance refers to a reduced biological response to glucocorticoid signaling despite the presence of endogenous or pharmacological glucocorticoids [13].

It is important to distinguish the evidence base underlying this mechanism from occupational-specific evidence. Most direct experimental evidence for stress-induced glucocorticoid resistance derives from general psychological-stress paradigms, human viral-challenge studies, and animal models rather than from longitudinal occupational cohorts. These findings therefore establish biological plausibility but should not be interpreted as direct demonstration of glucocorticoid resistance caused specifically by workplace stress.

In the context of chronic psychological stress, the concept is particularly relevant because persistent inflammation can occur even when cortisol production is preserved or increased [7,15]. The proposed mechanism involves reduced responsiveness of immune cells to the anti-inflammatory actions of endogenous glucocorticoids [7].

When glucocorticoid sensitivity decreases, cortisol becomes less effective at terminating inflammatory responses and suppressing the production of inflammatory mediators [7,9]. This mechanism was evaluated in two human viral-challenge studies conducted by Cohen and colleagues [7].

In the first study, 276 healthy adults were assessed for stressful life events, glucocorticoid receptor resistance, and subsequent susceptibility to experimentally induced upper respiratory infection [7]. Greater exposure to prolonged stressful events was associated with greater glucocorticoid receptor resistance [7]. Glucocorticoid receptor resistance was subsequently associated with an increased risk of developing a cold following viral exposure [7].

In the second study, glucocorticoid receptor resistance predicted increased local production of the pro-inflammatory cytokines IL-1β, IL-6, and TNF-α following infection with rhinovirus [7]. These findings support a model in which chronic stress increases disease susceptibility partly by impairing the capacity of glucocorticoids to regulate inflammatory responses [7,18].

Evidence from chronic psychological stress across species further supports an association between prolonged stress exposure and reduced glucocorticoid receptor sensitivity [15]. A systematic review found that chronic stress was reliably associated with downregulation of cellular glucocorticoid receptor sensitivity in animal and human studies [15]. Reduced glucocorticoid sensitivity was accompanied by increased peripheral inflammatory biomarkers, providing further evidence that receptor-level alterations may be more informative than cortisol concentration alone [15].

Several molecular processes may contribute to acquired glucocorticoid resistance [13]. These mechanisms include alterations in glucocorticoid receptor expression or function, changes in receptor-associated signaling pathways, excessive activation of mitogen-activated protein kinase, MAPK, pathways, and changes in transcriptional or epigenetic regulation [13].

Oxidative and nitrative stress can also impair glucocorticoid anti-inflammatory activity through mechanisms involving reduced histone deacetylase 2, HDAC2, activity and expression [19,20].

Excessive MAPK activation can interfere with GR-mediated signaling and decrease the anti-inflammatory effectiveness of glucocorticoids [13]. Importantly, chronic inflammation itself can promote glucocorticoid resistance, generating the possibility of a self-reinforcing cycle between inflammation and impaired glucocorticoid signaling [13].

Pro-inflammatory cytokines can also influence HPA-axis activity and interfere with its normal negative-feedback mechanisms, further connecting immune activation with neuroendocrine dysregulation [14]. Chronic stress may therefore produce a feed-forward process in which neuroendocrine activation promotes inflammatory signaling, inflammation alters HPA-axis regulation and glucocorticoid sensitivity, and reduced glucocorticoid sensitivity further limits control of inflammation [7,14].

Thus, chronic stress may produce an imbalance between pro-inflammatory adrenergic signaling and impaired glucocorticoid-mediated inflammatory control, favoring persistent low-grade inflammation [8,13]. This mechanism should not be interpreted as simple chronic hypercortisolemia, because altered receptor sensitivity and cortisol rhythmicity may be more relevant than absolute circulating cortisol concentrations [10,11,15].

This neuroendocrine–immune dysregulation provides the mechanistic basis for examining downstream inflammatory pathways, particularly NF-κB activation, cytokine production, immune-cell remodeling, oxidative stress, inflammaging, and stress-related transcriptional changes.

Overall, evidence for glucocorticoid resistance as a stress-related mechanism is biologically strong but remains largely indirect with respect to occupational exposure, because the most detailed mechanistic studies derive from general psychological-stress and experimental models rather than prospective worker cohorts.

## 4. Immunological Mechanisms Linking Stress to Inflammation

### 4.1. NF-κB Activation and Production of Pro-Inflammatory Cytokines

NF-κB represents a major transcriptional pathway linking chronic psychological stress to inflammatory gene expression. Human chronic-stress studies have demonstrated increased NF-κB-related transcriptional activity, including in circulating monocytes, alongside reduced glucocorticoid-responsive transcription. These findings complement the glucocorticoid-resistance mechanisms described in Section 3.3 and support a shift toward pro-inflammatory gene regulation during prolonged stress exposure [7,8]. 

Experimental human evidence also demonstrates that psychological stress can induce measurable changes in circulating inflammatory mediators [21]. A systematic review and meta-analysis found significant increases in circulating IL-1β, IL-6, TNF-α, and IL-10 following acute psychological stress [21]. The pooled standardized effect sizes were d = 0.66 for IL-1β, d = 0.35 for IL-6, d = 0.28 for TNF-α, and d = 0.69 for IL-10 [21]. 

The inflammatory response showed cytokine-specific temporal dynamics, with IL-6 increasing between approximately 40 and 120 min after stress exposure and reaching its largest pooled effect at approximately 90 min [21]. IL-1β increased earlier, whereas the significant increase in TNF-α was observed primarily between 31 and 50 min following the stressor [21]. In contrast, acute psychological stress did not produce a significant pooled increase in circulating CRP in this meta-analysis [21]. 

This distinction is relevant because CRP is synthesized predominantly by hepatocytes in response to upstream inflammatory mediators, particularly IL-6, and therefore has different kinetics from rapidly responsive cytokines [21]. 

Earlier meta-analytic evidence similarly demonstrated significant increases in circulating IL-6 and IL-1β following laboratory psychological stress [22].

In occupational settings, the evidence is less uniform than that obtained from controlled acute-stress experiments [2]. A systematic review of 56 studies concluded that psychosocial job stress was associated with measurable immune alterations, including increases in inflammatory markers, but also emphasized substantial methodological heterogeneity and insufficient evidence to establish definitive causal relationships [2]. 

Therefore, IL-6, TNF-α, IL-1β, and CRP should not be interpreted as interchangeable indicators of occupational stress, because their concentrations, kinetics, biological functions, and responsiveness to stress exposure differ [2,21].

### 4.2. Changes in Immune-Cell Function

The effects of psychological stress extend beyond circulating cytokine concentrations and include changes in the distribution and functional activity of innate and adaptive immune cells [2]. Occupational studies have reported stress-associated alterations in natural killer, NK, cells, T lymphocytes, lymphocyte subsets, and other immune parameters [2]. In a systematic review of psychosocial job stress and immunity, occupational exposures including high job demands, low job control, job strain, job dissatisfaction, effort–reward imbalance, overcommitment, burnout, unemployment, organizational downsizing, and economic recession were associated with measurable changes in immune parameters [2].

The reported alterations included reduced NK-cell activity, changes in NK-cell and T-cell subsets, changes in the CD4+/CD8+ ratio, and increased inflammatory markers [2]. The authors nevertheless concluded that additional prospective research was required before a causal relationship between psychosocial job stress and immune dysfunction could be established [2]. 

A systematic review focused specifically on healthcare professionals also found that the overall relationship between perceived occupational stress and biological parameters was inconsistent across studies [23]. Within that literature, comparatively consistent evidence was identified for associations involving NK cells and lymphocyte subpopulations [23]. However, only 16 studies met the inclusion criteria in that review, and considerable methodological heterogeneity limited the strength of the conclusions [23].

More recent evidence continues to support an effect of psychological stress on NK-cell biology [24]. A 2025 comprehensive review concluded that psychological stressors generally have negative effects on NK-cell activity across the examined literature, with prolonged stress producing greater reductions in NK-cell number and function [24].

Potential mechanisms of stress-associated NK-cell dysfunction include reduced release of perforin- and granzyme-containing secretory lysosomes, impaired formation of the NK-cell immunological synapse, changes in activating-receptor expression, and altered intracellular signaling [24]. The magnitude and direction of stress-related immune alterations depend on characteristics of the stressor, including its type, intensity, and duration, which limits simple comparisons between acute laboratory stress and chronic occupational exposure [24].

Monocytes represent another important cellular link between chronic psychosocial stress and inflammation because they are major contributors to innate inflammatory signaling and cytokine production [8]. Human chronic-stress studies have demonstrated transcriptional alterations within circulating monocytes that favor inflammatory gene-expression programs [8].

These findings suggest that chronic stress may alter immunity at several levels, including immune-cell distribution, cytotoxic activity, receptor sensitivity, and transcriptional programming [2,8].

### 4.3. Inflammaging and Associated Oxidative Stress

Inflammaging is defined as a state of chronic, low-grade inflammation that develops during aging and is associated with increased susceptibility to several age-related diseases [25]. Inflammaging has been implicated in cardiovascular disease, diabetes, rheumatoid arthritis, cancer, chronic obstructive pulmonary disease, and neurodegenerative disorders [25]. 

Oxidative stress is closely interconnected with inflammaging [25]. Reactive oxygen species, ROS, can oxidize and damage cellular proteins, lipids, and nucleic acids when their production exceeds cellular antioxidant capacity [25]. Excessive ROS generation can also promote inflammatory signaling and activate cellular death pathways [25]. Inflammation can itself promote additional ROS generation, creating reciprocal interactions between oxidative damage and inflammatory activity [25]. 

Mitochondrial dysfunction represents one potential contributor to this interaction because mitochondria are an important cellular source and target of reactive oxygen species [25]. Oxidative damage can consequently impair mitochondrial function, while dysfunctional mitochondria can further contribute to oxidative and inflammatory signaling [25]. 

The relationship between chronic psychological stress and inflammaging requires cautious interpretation because inflammaging is primarily an aging-related biological phenotype rather than a biomarker specific to occupational stress [25]. Nevertheless, chronic stress and inflammaging share several biological features, including persistent low-grade inflammatory signaling, altered immune regulation, oxidative stress, and pathways involved in cellular aging [25]. 

For occupational medicine, the relevant hypothesis is therefore that repeated psychosocial stress may interact with aging-related inflammatory processes rather than that workplace stress alone produces inflammaging [25]. This distinction is particularly relevant in aging workforces, in whom occupational psychosocial exposures coexist with age-related changes in immune regulation and increasing baseline risk of cardiovascular and metabolic disease [25]. 

### 4.4. Epigenetic Regulation and Stress-Induced Gene Expression

Evidence concerning stress-related epigenetic regulation derives predominantly from studies of general psychosocial adversity and chronic psychological stress rather than from occupational cohorts specifically. The mechanisms summarized below should therefore be interpreted as biologically plausible pathways relevant to occupational stress, not as established occupation-specific epigenetic signatures.

Chronic psychosocial adversity can become biologically embedded through changes in gene regulation without requiring alterations in the underlying DNA sequence [26].

Potential mechanisms include altered transcription-factor activity, chromatin remodeling, DNA methylation, histone modifications, and changes in non-coding RNA regulation [27].

DNA methylation involves the addition of methyl groups to DNA, most commonly at cytosine residues within CpG dinucleotides, and can influence transcriptional regulation depending on genomic context [28,29].

Stress-associated epigenetic changes have been investigated in genes involved in HPA-axis regulation, glucocorticoid signaling, immune function, and inflammatory responses [30]. 

Evidence linking work-related stress to DNA methylation remains limited and methodologically heterogeneous. Reviews examining daily-life stress, work stress, burnout, and epigenetic regulation have identified associations involving several candidate pathways, but the occupational literature remains comparatively small and predominantly observational. A systematic review of DNA methylation in stress, burnout, and depression identified only a small number of studies specifically examining work stress or burnout, emphasizing the need for longitudinal occupational research. More recent occupational evidence has also reported associations involving NR3C1 methylation in clinically selected workers, although cross-sectional designs do not permit causal or diagnostic interpretation. Accordingly, no DNA methylation pattern can currently be regarded as a validated or occupational-stress-specific biomarker [31,32,33].

The principal neuroendocrine and immune–inflammatory mechanisms linking chronic occupational stress to long-term disease are summarized in Figure 1.

### 4.5. Conserved Transcriptional Response to Adversity, CTRA

A particularly relevant framework for understanding the molecular effects of chronic psychosocial adversity is the Conserved Transcriptional Response to Adversity, CTRA [34]. Most CTRA evidence has been generated in studies of social adversity, isolation, caregiving, and other non-occupational psychosocial exposures; occupational evidence remains comparatively limited.

The CTRA is characterized by increased expression of pro-inflammatory genes together with decreased expression of genes involved in type I interferon antiviral responses and antibody-related immune functions [34]. This transcriptional profile has been investigated as a molecular correlate of chronic social adversity and threat-related psychological states [35]. 

In a longitudinal study of community-dwelling older adults, perceived social isolation was examined in relation to CTRA gene expression in peripheral blood [35]. The CTRA framework is relevant to stress immunology because it provides a transcriptional model linking psychosocial conditions with simultaneous enhancement of inflammatory gene expression and attenuation of selected antiviral immune programs [34,35].

One study recruited 114 employed Japanese men and reported an inverse association between dispositional optimism and CTRA gene expression. However, occupational stress was not the exposure under investigation. Recruitment from an employed population therefore does not establish an occupational-stress effect, and this study should be interpreted as evidence concerning psychosocial characteristics and CTRA expression in workers rather than as direct evidence of work-stress-induced transcriptional change [36].

CTRA should nevertheless be interpreted as a research framework rather than an established clinical biomarker of occupational stress [34]. Associations between adversity and CTRA are not universally observed across populations [37].

For example, a longitudinal analysis of 259 participants examining childhood adversity found no statistically significant association between the assessed adversity variables and CTRA gene expression [37]. This negative finding demonstrates that the relationship between psychosocial adversity and CTRA expression is context-dependent and should not be assumed to occur following every form of chronic stress exposure [37].

Current evidence therefore supports a multilevel model in which chronic stress can influence inflammation through altered neuroendocrine signaling, reduced glucocorticoid sensitivity, inflammatory transcription-factor activity, changes in leukocyte function, oxidative processes, and stress-responsive patterns of gene expression [2,8,21].

The strength of evidence differs substantially between these mechanisms, with particularly strong experimental support for stress-related cytokine responses and substantial observational evidence for altered immune-cell function, whereas epigenetic signatures and CTRA remain primarily research-level approaches rather than validated tools for occupational health surveillance [2,21,34].

The evidence supporting immune–inflammatory mechanisms is heterogeneous in directness: occupational data are available for circulating inflammatory markers and selected immune-cell outcomes, whereas detailed transcriptional, epigenetic, and CTRA mechanisms remain supported predominantly by non-occupational human or experimental studies.

## 5. Occupational Risk Factors and Stress Models

### 5.1. Job Demand–Control Model

The Job Demand–Control model, originally proposed by Robert Karasek, is one of the most widely used theoretical frameworks for evaluating psychosocial stress at work [38].

The model proposes that psychological strain is determined primarily by the interaction between psychological job demands and the degree of decision latitude or control available to the worker [39]. Psychological job demands refer to characteristics such as workload, time pressure, work intensity, and conflicting demands, whereas decision latitude reflects the worker’s ability to influence how and when tasks are performed and to use professional skills [39,40].

The combination of high psychological demands and low decision latitude is commonly defined as “job strain” [38]. The Job Content Questionnaire, JCQ, and related instruments are frequently used to operationalize the demand–control model in epidemiological research [38]. A systematic review identified 496 observational studies using the Job Demand–Control framework and demonstrated substantial heterogeneity in how high job strain was defined and measured [38]. This methodological variability is important because differences in instruments and exposure definitions can complicate direct comparison between occupational studies [38]. The demand–control framework has been used extensively to examine relationships between adverse psychosocial working conditions and cardiovascular, psychological, and other health outcomes [38].

Job strain has also been incorporated into large prospective studies examining cardiovascular disease risk associated with psychosocial occupational stress [1]. Evidence synthesized from large occupational cohorts indicates that job strain is associated with a modest increase in the risk of coronary heart disease compared with the absence of job strain [1].

The model was later extended by incorporating workplace social support, resulting in the Demand–Control–Support model [2].

The combination of high demands, low control, and low social support is often referred to as “iso-strain” and is considered a particularly adverse psychosocial work configuration [5]. In the PROspective Quebec Study on Work and Health, iso-strain was associated with a higher prevalence of an elevated inflammatory index among men [5]. Among men in that study, iso-strain was associated with a prevalence ratio of 1.42, 95% CI 1.06 to 1.90, for a high inflammatory index [5]. Among men younger than 65 years, the corresponding prevalence ratio was 2.00, 95% CI 1.37 to 2.92 [5]. These findings provide occupational evidence that psychosocial work organization, particularly the combination of high demand, limited control, and insufficient social support, may be associated with low-grade systemic inflammation [5].

### 5.2. Effort–Reward Imbalance Model

The Effort–Reward Imbalance, ERI, model developed by Johannes Siegrist represents a complementary framework for characterizing psychosocial stress at work [41].

The model is based on the principle of social reciprocity and proposes that work becomes particularly stressful when high occupational effort is not matched by adequate rewards [42]. Occupational rewards in the ERI framework include financial compensation, esteem or recognition, career opportunities, and employment security [42]. A persistent imbalance between high effort and low reward is considered a stressful condition capable of eliciting sustained psychobiological responses [42]. The ERI model additionally incorporates “overcommitment”, an intrinsic motivational pattern characterized by excessive work-related striving and difficulty withdrawing psychologically from occupational demands [42]. Overcommitment can increase vulnerability to an adverse effort–reward imbalance by maintaining high occupational investment even when rewards remain insufficient [42].

A systematic review of ERI research identified associations between effort–reward imbalance and a broad range of adverse health outcomes [42]. The strongest prospective evidence reviewed in that analysis concerned cardiovascular disease and depressive disorders [42]. Associations have also been reported between ERI and measures of poor self-rated health, reduced functioning, burnout, and several physiological outcomes [42].

A 2026 critical appraisal of three decades of ERI research concluded that a substantial body of prospective epidemiological evidence supports associations between effort–reward imbalance and increased health risks [41].

The ERI model has also been investigated in relation to biological markers potentially connecting occupational stress with chronic disease [43]. Studies based on the ERI model have examined cortisol, inflammatory biomarkers, immune parameters, blood pressure, heart-rate variability, lipid profiles, haemostatic markers, and metabolic abnormalities [43]. The evidence appears comparatively consistent for altered heart-rate variability, lipid abnormalities, and metabolic syndrome, whereas findings for cortisol, immune parameters, and inflammatory biomarkers have been more heterogeneous [43].

The Demand–Control and ERI models therefore capture different but complementary dimensions of occupational stress, with the former emphasizing workload and autonomy and the latter focusing on reciprocity between occupational effort and received rewards [38,42]. 

### 5.3. Occupational Burnout as a Distinct Occupational Phenotype

Burnout is closely related to chronic occupational stress but should not be used as a synonym for stress itself [44]. In ICD-11, the World Health Organization classifies burnout as an occupational phenomenon rather than a medical condition [44]. The ICD-11 conceptualizes burnout as a syndrome resulting from chronic workplace stress that has not been successfully managed [44]. According to ICD-11, burnout comprises three dimensions: energy depletion or exhaustion, increased mental distance from work or occupational negativism or cynicism, and reduced professional efficacy [44]. The WHO specifies that the concept of burnout refers specifically to the occupational context and should not be applied to experiences in unrelated areas of life [44].

Burnout has been associated with both work-related environmental factors and individual susceptibility factors [45]. Persistent burnout has been associated with impaired sleep, reduced quality of life, cognitive complaints, and increased prevalence or risk of several chronic disorders [45]. Cardiovascular and metabolic abnormalities have also been reported in the burnout literature, although the strength of causal evidence varies between outcomes [45].

Burnout is therefore relevant to occupational medicine as a specific manifestation of prolonged inadequately managed workplace stress, but its status differs from that of a formally classified medical disease [44,45].

### 5.4. Night and Shift Work

Night and rotating shift work constitute important occupational exposures because they combine work-related demands with disruption of sleep and circadian organization [46]. Shift work should not be interpreted as a purely psychosocial stress exposure. Its biological effects may arise from circadian misalignment, sleep restriction, altered meal timing, light exposure at night, and psychosocial demands, and these pathways cannot generally be separated in observational occupational studies.

A 2025 systematic review and meta-analysis identified 81 studies examining cardiovascular risk indicators and biomarkers among night-shift workers, including 14 cohort studies and 67 cross-sectional studies [46]. The review found moderate-confidence evidence for increased inflammation among night-shift workers [46]. The same review found evidence of dyslipidemia and impaired cardiac excitability in night-shift workers [46]. Dose-dependent associations were reported for several cardiovascular risk indicators, suggesting that both intensity and duration of night-shift exposure may contribute to risk [46].

Experimental occupational evidence also supports acute inflammatory changes following night work [47]. In a crossover study of 50 workers, inflammatory markers were measured after periods of daytime work and night work [47]. IL-6, CRP, total leukocyte counts, neutrophils, lymphocytes, and platelet counts were significantly higher after night work than after day work [47]. TNF-α also increased following night work, although this change did not reach statistical significance [47].

Cross-sectional data from the Japanese J-HOPE study have also examined IL-6 and high-sensitivity CRP in relation to shift and night work [48].

More recent occupational research has reported higher CRP-to-albumin ratios and neutrophil-to-lymphocyte ratios among long-term night-shift workers than among daytime workers [49]. In that study, the neutrophil-to-lymphocyte ratio was 2.19 in night workers compared with 1.84 in daytime workers, with *p* = 0.012 [49]. The CRP-to-albumin ratio was also higher in night workers, 0.44 versus 0.24, with *p* = 0.002 [49]. Because that study was cross-sectional and included 84 male workers, its findings demonstrate an association rather than proving that night work directly caused chronic inflammation [49].

In the population-based HUNT Study, shift work, CRP concentrations, and chronic musculoskeletal pain were evaluated among 23,223 occupationally active adults [50]. These data support the relevance of low-grade inflammation as one possible pathway connecting shift schedules with adverse health outcomes, while also illustrating that inflammation is only one component of the broader biological effects of circadian disruption [46,50].

### 5.5. High-Risk Occupations

Some occupational groups are exposed simultaneously to high psychological demands, acute traumatic events, organizational pressures, irregular schedules, sleep disruption, and physical hazards [51]. Law-enforcement personnel provide a useful model for studying the interaction between occupational stress and inflammatory cardiovascular risk [51].

In a study comparing 444 law-enforcement officers with 166 non-officers, investigators evaluated perceived stress, vital exhaustion, job strain, effort–reward imbalance, social support, and inflammatory mediators associated with atherosclerotic risk [51]. Law-enforcement officers had higher circulating concentrations of IL-1β, IL-6, IL-10, and TNF-α than non-officers in this study [51]. The officers unexpectedly had lower CRP and fibrinogen concentrations than the comparison group, illustrating that occupational stress does not produce a uniform pattern across all inflammatory markers [51].

A separate study of 71 law-enforcement officers also reported statistically significant relationships between several occupational stress measures and physiological or inflammatory variables [52]. These studies support the biological plausibility of stress-related immune and inflammatory alterations in high-risk occupations, but they do not establish that every high-risk profession has the same inflammatory profile [51,52,53]. 

Occupational category should therefore be considered together with specific exposures such as traumatic events, workload, shift schedules, perceived stress, organizational support, and duration of employment rather than being treated as an independent biological exposure [51].

### 5.6. Mobbing and Workplace Bullying

Workplace bullying represents a severe psychosocial occupational stressor with documented consequences for psychological and physical health [54]. Longitudinal evidence indicates that workers exposed to workplace bullying have an increased risk of subsequent mental health problems [54].

A systematic review and meta-analysis found an odds ratio of 1.68, 95% CI 1.35 to 2.09, for subsequent mental health problems among individuals exposed to workplace bullying [54]. The same analysis found an odds ratio of 1.77, 95% CI 1.41 to 2.22, for subsequent somatic symptoms [54]. Mental health problems also predicted subsequent workplace bullying in this analysis, suggesting that the relationship may be partly bidirectional [54].

A larger meta-analysis combining cross-sectional and longitudinal evidence found significant associations between workplace bullying and symptoms of depression, anxiety, and stress-related psychological complaints [55]. In cross-sectional analyses involving 115,783 participants, workplace bullying was associated with depression at r = 0.28, anxiety at r = 0.34, and stress-related psychological complaints at r = 0.37 [55]. Longitudinal data involving 54,450 participants also demonstrated an association between exposure to workplace bullying and subsequent mental health complaints [55].

Workplace bullying is additionally associated with occupational consequences such as sickness absence [56]. A systematic review and meta-analysis of prospective or registry-based studies found that workplace bullying was associated with a 58% higher odds of sickness absence, OR 1.58, 95% CI 1.39 to 1.79 [56].

Organizational characteristics are important determinants of workplace bullying risk [57]. A systematic review of 51 studies identified associations between bullying and organizational factors including authoritarian or laissez-faire leadership, role conflict, role ambiguity, high workload, work pressure, unclear duties, and certain forms of work organization [57].

In healthcare workers, workplace bullying has also been associated with higher job stress and poorer professional quality of life [58].

The evidence linking workplace bullying specifically to inflammatory biomarkers is considerably less developed than the evidence linking bullying to psychological morbidity, somatic symptoms, and occupational outcomes [54,55].

For this reason, workplace bullying can reasonably be considered a chronic psychosocial stressor capable of engaging the neuroendocrine pathways described earlier, but a specific inflammatory signature of occupational bullying has not yet been established [2,54].

### 5.7. Integration of Occupational Stress Models with Immune–Inflammatory Mechanisms

The Job Demand–Control and Effort–Reward Imbalance models provide complementary approaches for identifying chronic psychosocial exposures that may repeatedly activate stress-response systems [38,42].

Night work adds circadian and sleep disruption to these psychosocial pathways and has comparatively direct evidence linking occupational exposure with inflammatory alterations [46].

High-risk occupations may combine multiple exposures, including acute stressors, chronic organizational stress, disturbed sleep, and effort–reward imbalance, making attribution to a single biological pathway difficult [51].

Workplace bullying represents another persistent psychosocial stressor with strong evidence for adverse mental and occupational outcomes, although direct evidence for specific immune–inflammatory consequences remains comparatively limited [54,55].

Taken together, these occupational exposures provide the epidemiological context in which repeated HPA-axis activation, sympathetic signaling, impaired glucocorticoid regulation, inflammatory transcription, and immune-cell alterations may develop and contribute to long-term disease risk [2,15].

Among occupational exposures, the strongest direct evidence concerns established psychosocial models such as job strain and effort–reward imbalance, while night and shift work additionally involve circadian and sleep-related pathways that should not be interpreted as purely psychosocial stress effects.

## 6. Clinical Consequences

### 6.1. Cardiovascular Disease and Inflammation-Mediated Atherosclerosis

Chronic psychosocial stress at work has been associated with an increased risk of incident cardiovascular disease, particularly coronary heart disease and stroke [1].

A review integrating data from more than 600,000 workers across 27 cohort studies conducted in Europe, the United States, and Japan found that job strain and long working hours were associated with approximately 10% to 40% higher risks of incident coronary heart disease or stroke compared with the absence of these exposures [1].

The association between occupational stress and coronary heart disease has also been demonstrated using individual-participant data [59,60]. In a collaborative meta-analysis of 13 European cohort studies, 197,473 participants without coronary heart disease were followed for a mean of 7.5 years [59]. Job strain was reported by 30,214 participants, corresponding to approximately 15% of the study population [59]. During 1.49 million person-years of follow-up, 2358 incident coronary heart disease events were recorded [59].

After adjustment for age and sex, job strain was associated with a hazard ratio of 1.23, 95% CI 1.10 to 1.37, for incident coronary heart disease [59]. The association remained after excluding coronary events occurring within the first three and five years of follow-up, which reduced the likelihood that reverse causation from pre-existing cardiovascular disease fully explained the result [59]. The population-attributable risk associated with job strain was estimated at 3.4% in this analysis [59].

Effort–reward imbalance has independently been associated with coronary heart disease [61]. A multicohort analysis of 90,164 workers from 11 European prospective cohorts found a hazard ratio of 1.16, 95% CI 1.00 to 1.35, for coronary heart disease among workers with effort–reward imbalance compared with those without imbalance [61]. Participants simultaneously exposed to effort–reward imbalance and job strain had a hazard ratio of 1.41, 95% CI 1.12 to 1.76, compared with workers exposed to neither occupational stressor [61]. This finding suggests that different dimensions of psychosocial work stress may have cumulative cardiovascular effects [61].

Part of the cardiovascular association may be mediated through conventional behavioral and metabolic risk factors [62]. An individual-participant meta-analysis of 47,045 workers found that job strain was associated with diabetes, smoking, physical inactivity, obesity, and a higher overall Framingham cardiovascular risk score [62]. Compared with workers without job strain, workers reporting job strain had higher odds of diabetes, OR 1.29, 95% CI 1.11 to 1.51 [62].

Job strain was also associated with physical inactivity, OR 1.34, 95% CI 1.26 to 1.41, smoking, OR 1.14, 95% CI 1.08 to 1.20, and obesity, OR 1.12, 95% CI 1.04 to 1.20 [62]. By contrast, the same analysis did not demonstrate clear associations between job strain and clinic blood pressure or blood lipid concentrations [62].

Inflammation represents an additional plausible pathway linking chronic occupational stress with atherosclerotic cardiovascular disease [63]. Psychosocial stressors at work may contribute to low-grade systemic inflammation through interactions between immune pathways and neuroendocrine stress systems [63].

Occupational studies have reported associations between work-related stress and inflammatory mediators including IL-6, TNF-α, IL-1β, and CRP, although results vary across populations and biomarkers [2].

Persistent activation of NF-κB-related inflammatory transcription and impaired glucocorticoid control of inflammation provide plausible mechanisms through which chronic stress could contribute to endothelial dysfunction and vascular inflammation [7,8].

Inflammation is a fundamental component of atherosclerotic plaque initiation, progression, destabilization, and thrombosis, making chronic low-grade inflammatory activation biologically relevant to cardiovascular risk.

Nevertheless, inflammation should be interpreted as one mediator among several rather than as the sole explanation for the association between occupational stress and cardiovascular disease [1].

Autonomic dysregulation, disturbed sleep, smoking, reduced physical activity, obesity, glucose dysregulation, and other behavioral or metabolic factors may operate in parallel with inflammatory mechanisms [62].

### 6.2. Metabolic Syndrome, Insulin Resistance, and Type 2 Diabetes

Chronic psychological stress has been investigated as a potential contributor to metabolic syndrome and its individual components [6]. A systematic review and meta-analysis found that individuals classified in high psychological-stress groups had 45% higher adjusted odds of metabolic syndrome than those in low-stress groups, with an OR of 1.45, 95% CI 1.21 to 1.74 [6]. When analyses were stratified according to the source of psychological stress, occupational stress produced the strongest pooled association, with an OR of 1.69, 95% CI 1.18 to 2.42 [6]. However, the occupational-stress estimate in this meta-analysis was derived primarily from cross-sectional studies, limiting causal interpretation [6].

Earlier prospective evidence also suggested an association between chronic psychosocial stress and subsequent development of metabolic syndrome, although study designs and exposure definitions were heterogeneous [64]. A systematic review of 39 prospective studies found general support for an association between chronic psychosocial stress and development of metabolic syndrome, while results for specific occupational stress exposures were less consistent [64].

More recent prospective occupational evidence strengthens the link between workplace stress, insulin resistance, and type 2 diabetes. A prospective community cohort in Beijing included 6109 participants who were assessed for occupational stress using the Copenhagen Psychosocial Questionnaire and followed from 2015 to 2021. At baseline, higher scores across the occupational-stress dimensions were significantly associated with insulin resistance. During a mean follow-up of 5.63 years, 732 participants developed type 2 diabetes. Higher scores for work demands, job insecurity, job satisfaction-related measures, and total occupational-stress scores were significantly associated with incident type 2 diabetes. Mediation analyses in this cohort suggested that plasma cortisol was involved in the pathway between perceived occupational stress and incident type 2 diabetes [65].

HOMA-IR mediated approximately 37.1% of the relationship between cortisol and incident type 2 diabetes in the reported model. Blood pressure, triglycerides, urea, and uric acid also showed smaller statistically significant mediation effects in the same analysis. These findings provide prospective evidence supporting a pathway from occupational psychosocial stress through neuroendocrine and metabolic dysregulation to insulin resistance and diabetes [65].

A 2026 systematic review and meta-analysis examining perceived stress and metabolic parameters included 30 studies and 12,491 participants [66].

Higher perceived stress was positively associated with insulin resistance measured using HOMA-IR, with a meta-regression coefficient of β = 0.463, 95% CI 0.159 to 0.767 [66]. The same meta-analysis did not find significant pooled associations between perceived stress and most traditional metabolic parameters, including body mass index, waist circumference, blood pressure, fasting glucose, and triglycerides [66]. The authors emphasized that the HOMA-IR association was exploratory, heterogeneous, and sensitive to individual studies, so causal conclusions remain premature [66].

Shift work represents an additional occupational pathway relevant to metabolic disease. A systematic review of ten studies examining shift work and metabolic syndrome found positive associations in eight studies after adjustment for sociodemographic and behavioral factors [67]. However, differences in metabolic-syndrome definitions, shift-work classifications, and adjustment for sleep prevented a definitive conclusion about causality [67].

The metabolic effects of chronic stress are likely to involve interactions between HPA-axis activation, cortisol exposure, sympathetic activity, inflammatory signaling, insulin sensitivity, adipose tissue biology, sleep, and health behaviors [65].

Pro-inflammatory cytokines may interfere with insulin-signaling pathways, providing a biological link between chronic low-grade inflammation and insulin resistance. The occupational evidence therefore supports an association between psychosocial stress and metabolic dysfunction, but the strength of evidence varies according to the outcome, with particularly relevant emerging evidence for insulin resistance and incident type 2 diabetes [65,66].

These mediation estimates should be interpreted as statistical pathway models rather than definitive evidence of biological mediation, because causal mediation requires appropriate temporal ordering and strong assumptions regarding unmeasured confounding.

### 6.3. Chronic Inflammation, Neuroendocrine Regulation, and Skeletal Health—Indirect, Hypothesis-Generating Evidence

Beyond cardiovascular and metabolic consequences, persistent immune–inflammatory activation may also have implications for skeletal health. In a 12-month cross-sectional study of 120 adults with moderate-to-severe atopic dermatitis and 120 healthy controls, osteoporosis was more frequent in patients with atopic dermatitis than in controls (25% vs. 10%), while osteopenia was also more prevalent (40% vs. 22%); longer disease duration, greater disease severity, and systemic corticosteroid exposure were associated with less favorable skeletal outcomes [68]. Two additional case reports of long-standing atopic dermatitis and osteoporosis highlighted a clinical phenotype in which chronic inflammatory activity, corticosteroid exposure, and reduced physical activity may coexist and collectively contribute to skeletal vulnerability [69,70]. The possible involvement of type 2 cytokine pathways in bone remodeling has also been raised by individual observations of osteoporosis during IL-4/IL-13-targeted therapy [71,72]. However, these reports are hypothesis-generating and do not establish a causal relationship between IL-4/IL-13 blockade and osteoporosis. Collectively, these findings support a multifactorial relationship between chronic inflammation and skeletal health, while emphasizing the need for prospective longitudinal studies before specific causal or therapeutic conclusions can be drawn.

Bone is increasingly recognized as a metabolically active endocrine organ rather than solely as a structural tissue, with bone-derived mediators participating in systemic metabolic and endocrine regulation [73]. Skeletal remodeling is integrated within a broader neuroendocrine network involving hypothalamic, pituitary, thyroid, gonadal, and adipose-derived signals, including ACTH, TSH, growth hormone, sex hormones, leptin, adiponectin, and neuropeptide Y [74]. NPY may provide an additional link between neural and endocrine regulation of skeletal homeostasis, as its signaling can modulate osteoblast activity and interact with sex-hormone-dependent pathways controlling bone mass [75]. These observations broaden the concept of skeletal homeostasis to include coordinated neural, endocrine, metabolic, and immune regulation.

Endocrine disorders may further modify skeletal vulnerability. In patients with thyrotoxicosis-related osteoporosis, excess thyroid hormone activity has been associated with accelerated bone turnover, affecting both bone formation and resorption, with predominance of the resorptive component [76]. Clinical evaluation of patients with different forms of thyrotoxicosis has similarly documented reduced bone mineral density and involvement of both trabecular and cortical bone [77]. Lifestyle exposures must also be considered, as tobacco use has been associated with impaired skeletal health and may represent an important confounder when evaluating relationships between chronic psychosocial stress and bone outcomes [78].

Taken together, these observations indicate that skeletal homeostasis is influenced by overlapping inflammatory, neuroendocrine, hormonal, behavioral, and treatment-related factors. Although these data do not demonstrate that occupational stress directly causes osteoporosis, they identify biologically plausible pathways and important confounders that should be considered in future longitudinal studies examining chronic workplace stress and skeletal health.

The relationship between chronic stress and autoimmune disease is biologically plausible because the HPA axis, sympathetic nervous system, glucocorticoid signaling, and inflammatory cytokines directly regulate innate and adaptive immune responses. Stress-related alterations in immune regulation may affect lymphocyte function, cytokine balance, inflammatory transcription, and glucocorticoid sensitivity, mechanisms that could influence autoimmune disease susceptibility or activity [2].

Large population-based studies have identified associations between severe stress-related disorders and subsequent autoimmune disease. A Swedish nationwide cohort study included 106,464 individuals with stress-related disorders, more than one million matched unexposed individuals, and 126,652 unaffected full siblings [79]. Stress-related disorders were associated with an increased subsequent incidence of autoimmune disease compared with both population controls and sibling controls [79]. The study evaluated 41 autoimmune diseases and included stress-related diagnoses such as post-traumatic stress disorder, acute stress reaction, and adjustment disorder [79]. These findings support an association between severe stress exposure and autoimmunity but do not establish occupational stress as a direct cause of autoimmune disease [79].

Stress may also influence the clinical course of established autoimmune diseases. In multiple sclerosis, psychological stress has been evaluated in relation to disease onset, relapse risk, and disability progression [80]. A 2024 systematic review and meta-analysis found evidence supporting an association between stressful life events or perceived stress and multiple-sclerosis activity, particularly relapse risk [80]. Interpretation was limited by heterogeneity in stress assessment, study design, and disease-related outcomes [80].

Psychological stress has also been examined extensively in rheumatoid arthritis [81]. A systematic review found considerable heterogeneity in how stress was assessed in rheumatoid arthritis, including social, work-related, and role-related stressors [81].

Prospective data suggest that short-term psychological stress and worrying may precede fluctuations in fatigue, pain, swollen-joint count, and self-reported disease activity in patients with rheumatoid arthritis [82]. In a six-month prospective study of 80 rheumatoid-arthritis patients, daily stressors predicted increased fatigue during the following month [82]. Worrying predicted subsequent increases in self-reported disease activity, swollen-joint count, and pain [82].

### 6.4. Depression, Anxiety, and Shared Inflammatory Mechanisms

Adverse psychosocial working conditions have been associated with depressive symptoms, psychological distress, and depressive disorders, but these outcomes should not be treated as interchangeable and causal interpretation remains uncertain. Mikkelsen et al. reviewed 54 studies addressing 19 psychosocial occupational exposures and 11 different measures of depression. Although most summary estimates were above unity, substantial heterogeneity, possible diagnostic misclassification, inadequate control of confounding, and common-method bias limited causal inference. The authors concluded that the available evidence did not permit confident judgments that the assessed psychosocial work exposures caused depressive episodes or recurrent depressive disorders [83].

Workplace bullying has particularly consistent associations with subsequent psychological morbidity [54]. Longitudinal meta-analysis found an OR of 1.68, 95% CI 1.35 to 2.09, for subsequent mental-health problems among workers exposed to workplace bullying [54].

Depression is also relevant to the immunological framework of chronic occupational stress because inflammatory abnormalities are observed in a proportion of individuals with depressive illness [84]. A 2023 systematic review and meta-analysis of 64 studies found evidence of increased inflammatory activity in clinically significant depression [84].

The same meta-analysis found more tentative evidence for inflammatory alterations in anxiety disorders and post-traumatic stress disorder. The inflammatory profiles differed between depression, anxiety, and PTSD, indicating that there is no single inflammatory signature shared uniformly across these conditions [84].

Meta-analytic evidence has repeatedly identified elevations in CRP and IL-6 among individuals with major depressive disorder.

These inflammatory abnormalities may reflect multiple interacting processes, including psychosocial stress, altered glucocorticoid sensitivity, obesity, sleep disruption, physical inactivity, smoking, metabolic disease, and the depressive disorder itself. Chronic stress may facilitate inflammatory signaling through activation of sympathetic pathways and attenuation of glucocorticoid-mediated inflammatory control [7].

Inflammatory biomarkers cannot currently diagnose occupational depression or distinguish a work-related depressive disorder from depression arising in other contexts [84]. Increased inflammatory markers should therefore be interpreted as one possible biological phenotype within heterogeneous depressive and anxiety disorders rather than as universal markers of psychiatric disease.

### 6.5. Interaction Between Inflammation, Sickness Behavior, and Work Ability

Low-grade systemic inflammation may also influence functional outcomes relevant to occupational medicine [63]. Inflammatory signaling can produce behavioral and somatic responses including fatigue, reduced activity, sleep alteration, impaired concentration, and diminished motivation, phenomena commonly grouped under the concept of sickness behavior [63].

These symptoms overlap substantially with complaints frequently reported by workers experiencing chronic stress or burnout, including fatigue, poor concentration, sleep problems, and reduced work capacity. The overlap does not demonstrate that inflammatory activation is responsible for these symptoms in individual workers. It does, however, provide a mechanistic explanation for why sustained inflammatory activation could contribute to reduced occupational functioning when combined with psychological distress and inadequate recovery [63].

Work-related low-grade inflammation may therefore be relevant to productivity, sickness absence, and work ability as well as to long-term disease risk [63].

### 6.6. Clinical Interpretation in Occupational Medicine

The clinical consequences associated with chronic occupational stress are heterogeneous and should not be interpreted as a single “stress disease”.

Cardiovascular disease currently has some of the strongest prospective occupational evidence, with associations demonstrated for both job strain and effort–reward imbalance [59,61].

Metabolic evidence is increasingly supported by prospective data linking occupational stress with insulin resistance and incident type 2 diabetes [65].

The association with metabolic syndrome is supported by meta-analysis, but occupational-specific longitudinal evidence remains less consistent than the evidence for coronary heart disease [6,64].

Associations between adverse psychosocial working conditions and mental-health outcomes are repeatedly observed in occupational epidemiology, but the strength of causal inference depends on study design and outcome definition, and evidence based on depressive symptoms or psychological distress should not be interpreted as equivalent to evidence for clinically diagnosed depressive disorder [83].

The progression from occupational psychosocial exposures to biological dysregulation and subsequent clinical outcomes, together with relevant moderating and feedback mechanisms, is illustrated in Figure 2.

Autoimmune outcomes have substantial biological plausibility and epidemiological support for severe stress in general, but direct evidence specific to chronic occupational stress remains substantially weaker. Inflammation provides a shared mechanistic pathway across several of these outcomes but does not fully explain their development.

A more comprehensive model includes simultaneous interactions among HPA-axis dysregulation, sympathetic activation, glucocorticoid resistance, inflammatory signaling, disturbed sleep, health behavior, metabolic abnormalities, and individual susceptibility.

Clinical assessment in occupational medicine should therefore focus on identifiable psychosocial exposure together with the worker’s cardiovascular, metabolic, psychological, sleep-related, and inflammatory context rather than attempting to attribute nonspecific symptoms or laboratory abnormalities to stress alone. Clinical evidence is strongest for cardiovascular and selected metabolic and mental-health associations, whereas autoimmune and skeletal outcomes remain substantially more indirect with respect to occupational exposure.

## 7. Biomarkers for Occupational Health Surveillance

### 7.1. C-Reactive Protein and Interleukin-6

C-reactive protein, CRP, and interleukin-6, IL-6, are among the inflammatory biomarkers most frequently investigated in studies examining the biological effects of chronic psychosocial stress. CRP is widely used as a marker of systemic low-grade inflammation, while IL-6 is a pro-inflammatory cytokine involved in the regulation of hepatic acute-phase responses and several immune and metabolic processes [5].

The most directly relevant prospective synthesis was provided by Eguchi et al., who conducted a systematic review and meta-analysis of 11 longitudinal or prospective studies examining work-related psychosocial factors and inflammatory markers among workers. The pooled association was statistically significant but small (β = 0.014, 95% CI 0.005–0.023), and a clear marker-specific association was observed only for IL-6. Importantly, all included studies were judged to have serious risk of bias. These findings therefore support, at most, a weak prospective association between adverse psychosocial working conditions and inflammatory activity, with more consistent evidence for IL-6 than for CRP or TNF-α, while the overall certainty of the occupational evidence remains limited [85].

Their relevance to occupational medicine derives from evidence that psychosocial stressors at work may be associated with persistent low-grade inflammatory activity. The PROspective Quebec Study on Work and Health evaluated CRP and IL-6 in a cohort originally comprising 9188 white-collar workers who were followed over several decades [5].

At the biomarker assessment, 2557 participants were selected for standardized serum measurements of CRP and IL-6. Psychosocial exposures were evaluated using recognized occupational stress models, including the Demand–Control–Support model and the Effort–Reward Imbalance model [5].

Among men, exposure to iso-strain was associated with a higher prevalence of an elevated combined inflammatory index, with a prevalence ratio of 1.42, 95% CI 1.06 to 1.90. Among men younger than 65 years, the corresponding prevalence ratio was 2.00, 95% CI 1.37 to 2.92 [5]. Because statistical significance within one subgroup does not by itself demonstrate a statistically significant difference between subgroups, these sex- and age-stratified findings should not be interpreted as evidence of effect modification unless supported by formal interaction testing.

Associations with inflammatory biomarkers were also observed in selected groups exposed to effort–reward imbalance, low occupational reward, or moderate workplace social support. The authors nevertheless concluded that further studies are required to confirm these relationships and clarify differences between men and women [5].

Evidence from the Whitehall II cohort similarly indicates that relationships between workplace stress, inflammatory markers, and psychological symptoms may differ according to sex. In that study, which included 2528 participants, workplace stress, depressive symptoms, IL-6, and CRP were evaluated concurrently [86].

A cross-sectional statistical mediation model suggested an indirect association involving depressive symptoms among women. Because workplace stress, depressive symptoms, and inflammatory markers were assessed concurrently, this analysis does not establish temporal mediation or a causal sequence from workplace stress through depression to IL-6 [86].

CRP and IL-6 are therefore useful research biomarkers for evaluating low-grade inflammation associated with psychosocial occupational exposures, but neither marker is specific for occupational stress [5].

Both biomarkers may be influenced by chronic inflammatory diseases, acute infections, obesity, metabolic abnormalities, smoking, medication use, age, and other biological or behavioral factors that must be considered when interpreting stress-related associations. Consequently, an elevated CRP or IL-6 concentration in an individual worker cannot establish that workplace stress is the cause of the inflammatory alteration [5].

### 7.2. Salivary Cortisol

Salivary cortisol is one of the most extensively investigated non-invasive biomarkers of HPA-axis activity in occupational stress research [87,88].

Salivary cortisol reflects the unbound fraction of circulating cortisol and can be collected repeatedly during normal daily activities without venipuncture, which makes it attractive for field studies in workers [87,88].

Interpretation of salivary cortisol requires repeated measurements because cortisol secretion follows a marked circadian rhythm rather than remaining stable throughout the day. Cortisol concentrations are normally highest in the morning and decrease progressively toward the evening [89].

Occupational studies therefore commonly assess indices such as the cortisol awakening response, diurnal cortisol slope, individual sampling points, or total cortisol output across the day [87,88].

In a study of 104 call-centre workers, seven salivary cortisol measurements were obtained across two workdays and one leisure day to assess cortisol output and the cortisol awakening response. In that study, cortisol secretion during the awakening period was positively associated with measures of job strain [87,88].

Occupational shift work has also been associated with alterations in salivary cortisol profiles. In a study of 55 automobile-industry workers performing early shifts, salivary cortisol concentrations were higher during the working day than during a day off, particularly in the evening. The workers also showed a more attenuated decline in cortisol across the working day [89].

More recent occupational research combining work-stress diaries with salivary cortisol measurements found that cortisol concentrations were significantly associated with reported subjective stress levels in the pooled workforce sample [90].

However, salivary cortisol does not show a consistent relationship with every occupational psychosocial stressor. In a study of 341 workers from 34 workplaces in Quebec, five saliva samples per day were collected across two working days and one day off [91].

Skill utilization and job insecurity were associated with salivary cortisol intensity, whereas psychological demands and job insecurity were associated with depressive symptoms. Salivary cortisol did not mediate the relationship between the assessed work-related stressors and depression in that study [91].

These findings illustrate a major limitation of salivary cortisol as a screening biomarker, namely that the relationship between psychosocial stress exposure and HPA-axis activity is neither simple nor uniform across workers [87,91].

Sampling time, awakening time, sleep duration and quality, work schedule, sex, age, physical activity, and adherence to the sampling protocol can influence salivary cortisol measurements and therefore require careful control in occupational studies [87,88,92].

A single salivary cortisol measurement is consequently insufficient to characterize chronic occupational stress reliably because it may predominantly reflect the circadian phase and short-term physiological state at the time of sampling [87,88].

### 7.3. Hair Cortisol as a Marker of Longer-Term HPA-Axis Activity

Hair cortisol concentration has been proposed as an alternative method for assessing cumulative cortisol exposure over longer periods than those captured by saliva or blood sampling [93].

Hair cortisol analysis offers the possibility of retrospectively assessing longer-term HPA-axis activity and is therefore potentially relevant to chronic rather than momentary stress exposure [94].

Because scalp hair grows progressively, sequential hair segments can provide information on cumulative cortisol exposure over preceding periods, although interpretation depends on the analytical and sampling methodology used. Hair cortisol is attractive for occupational surveillance because collection is non-invasive and does not require repeated daily sampling by the employee [93].

A scoping review specifically evaluating hair cortisol in occupational healthcare identified five longitudinal studies examining work-related stressors, perceived stress, or mental-health outcomes among workers. Two of these longitudinal studies found that increases in work-related stressors were associated with higher hair cortisol concentrations [93].

More recent quantitative evidence continues to show variability in the relationship between hair cortisol-related measures and perceived chronic stress. A 2025 systematic review and meta-analysis examined hair cortisol and dehydroepiandrosterone, DHEA, in adults exposed to chronic stress for at least four weeks [95].

Eight studies met the inclusion criteria, comprising five cross-sectional studies and three cohort studies. A meta-analysis of studies using validated perceived-stress scales found no significant correlation between the hair cortisol-to-DHEA ratio and perceived stress, with ρ = 0.09, 95% CI −0.07 to 0.26. By contrast, an analysis comparing high-stress and low-stress groups found a moderate pooled difference, Hedges’ g = 0.69, 95% CI 0.14 to 1.24, although heterogeneity was substantial, I^2^ = 77.17% [95].

These findings reinforce the conclusion that hair cortisol-related measures have biological and research value but are not yet sufficiently standardized or consistent to serve as standalone diagnostic tests for chronic occupational stress [93,95].

The main inflammatory and neuroendocrine biomarkers investigated in relation to chronic occupational stress, together with their biological relevance and principal limitations, are summarized in Table 1.

### 7.4. Intended Use and Clinical Validity of Candidate Biomarkers

The clinical value of a biomarker depends on its intended use. Four distinct applications should be considered separately: identification of occupational psychosocial exposure, diagnosis of a clinical condition, prediction of future disease, and monitoring of change over time. At present, CRP, IL-6, salivary cortisol, and hair cortisol have not demonstrated sufficient occupational-stress specificity for exposure identification, and no validated diagnostic-accuracy framework has established sensitivity, specificity, or clinically actionable thresholds for diagnosing occupational stress. Evidence that these biomarkers improve prediction of clinical outcomes beyond conventional risk factors, occupational history, and validated psychosocial assessment is also lacking. Longitudinal biomarker measurements may be useful for research on within-person change, but clinically validated monitoring thresholds and evidence that biomarker-guided management improves outcomes have not been established.

The potential value of biological markers in occupational medicine lies in complementing, rather than replacing, assessment of psychosocial working conditions and workers’ clinical symptoms. Current occupational stress assessment relies predominantly on validated questionnaires, clinical evaluation, work-organization analysis, and identification of psychosocial hazards. A biomarker-based approach could theoretically provide additional objective information regarding physiological stress responses, particularly when repeated measurements are available [93].

However, none of the biomarkers discussed above currently provides a biological signature that is specific for occupational psychosocial stress [5,93].

CRP and IL-6 primarily indicate inflammatory activity and cannot identify the source of that inflammation [5].

Salivary cortisol is highly sensitive to sampling time and short-term physiological conditions and therefore requires repeated, carefully standardized measurements [87,88].

Hair cortisol potentially reflects longer-term HPA-axis activity but currently lacks sufficient consistency across occupational studies to define clinically validated thresholds for chronic work stress. Biomarker measurements should therefore not currently be used in isolation to classify an individual employee as chronically occupationally stressed. Their more defensible present role is in research, longitudinal monitoring, evaluation of intervention effects, and potentially multimodal risk assessment when combined with validated psychosocial and clinical measures [93].

Evidence that biomarkers can respond to occupational stress-reduction interventions supports this potential monitoring role. In a randomized trial involving 30 university employees with high stress levels, an eight-week mindfulness intervention was associated with reductions in hair cortisol, perceived stress, and anxiety compared with a waiting-list control group [96].

The small sample size of that trial limits generalization of the findings to broader occupational populations. The study nevertheless illustrates how longitudinal biomarker measurements may be more informative for evaluating within-person changes following an occupational intervention than a single measurement used for diagnostic screening [96].

### 7.5. Practical Implications for Occupational Health Surveillance

Accordingly, routine occupational-health assessment should continue to rely on validated psychosocial assessment, occupational history, clinical evaluation, and established disease-specific investigations. The biomarkers reviewed here should currently be regarded as investigational measures for occupational-stress assessment rather than established adjunctive surveillance tests.

Any future implementation of biological stress monitoring in occupational settings would require clear informed consent, confidentiality, predefined clinical purpose, independent interpretation by occupational-health professionals, and safeguards against inappropriate employment decisions based on nonspecific or insufficiently validated biological measurements.

From a surveillance perspective, the strongest current approach is a multimodal assessment combining validated psychosocial questionnaires, occupational history, clinical evaluation, and conventional cardiovascular or metabolic risk assessment, with biomarkers used selectively when clinically or scientifically justified [5,93].

CRP may be particularly practical because it is already widely available in clinical laboratories, but its lack of specificity substantially limits its value as a direct biomarker of occupational stress. IL-6 may provide more direct information on inflammatory signaling but is less suited to routine occupational screening because its interpretation remains strongly context-dependent and the occupational evidence is still heterogeneous [5].

Salivary cortisol is useful when the objective is to characterize diurnal HPA-axis function, but reliable assessment requires strict control of sampling schedules and relevant confounders [87,88].

Hair cortisol is potentially better aligned with the concept of chronic occupational stress because it integrates cortisol exposure over a longer period, but its occupational-health application remains investigational [93].

The absence of validated occupational reference intervals, universally accepted cut-off values, and sufficiently high specificity currently prevents CRP, IL-6, salivary cortisol, or hair cortisol from functioning as standalone screening tests for chronic workplace stress [5,93].

Future occupational surveillance research should therefore determine whether combinations of psychosocial exposure measures, clinical characteristics, inflammatory biomarkers, and longitudinal HPA-axis markers provide greater predictive value than any individual biomarker [93].

From a diagnostic perspective, the current biomarker evidence is insufficient to support occupational-stress diagnosis, individual risk classification, or routine surveillance, because analytical and clinical validity, specificity, and incremental value over conventional assessment have not been demonstrated.

## 8. Conclusions

This review has several limitations. First, although a structured literature search was performed, the review was narrative rather than systematic, and PubMed/MEDLINE was used as the principal bibliographic database; relevant publications indexed elsewhere may therefore have been missed. Second, occupational psychosocial exposures were defined using heterogeneous instruments and theoretical models, limiting direct comparison across studies. Third, several mechanistic pathways discussed in this review are supported primarily by experimental, general psychological-stress, caregiving, clinical, or preclinical studies rather than by direct occupational evidence. Fourth, repeated analyses from major occupational cohorts may contribute to apparent replication without representing fully independent populations. Fifth, candidate inflammatory, neuroendocrine, transcriptional, and epigenetic biomarkers lack sufficient validation for occupational diagnostic or surveillance purposes. Finally, selected conference-abstract evidence was included only for contextual and hypothesis-generating purposes and should not be assigned the same evidential weight as full peer-reviewed prospective studies.

Chronic occupational stress is associated with neuroendocrine and immune–inflammatory dysregulation that may contribute to cardiovascular, metabolic, psychiatric, and, potentially, autoimmune disease. Repeated activation of the HPA axis and sympathetic nervous system, impaired glucocorticoid signaling, pro-inflammatory cytokine activity, immune-cell alterations, oxidative stress, and stress-related transcriptional changes provide plausible biological mechanisms linking workplace stress to long-term health outcomes. Current biomarkers, including CRP, IL-6, salivary cortisol, and hair cortisol, remain useful mainly for research and longitudinal monitoring because none is sufficiently specific for routine diagnosis or screening of occupational stress. Preventive implications should primarily be interpreted in relation to the identification of and reduction in adverse psychosocial occupational exposures, while evidence linking individual stress-reduction interventions to clinically meaningful immune–inflammatory outcomes remains limited. Future longitudinal studies using standardized exposure assessment and repeated biomarker measurements are required to clarify causality and determine whether biological markers can add meaningful value to occupational health surveillance.

## Figures and Tables

**Figure 1:**
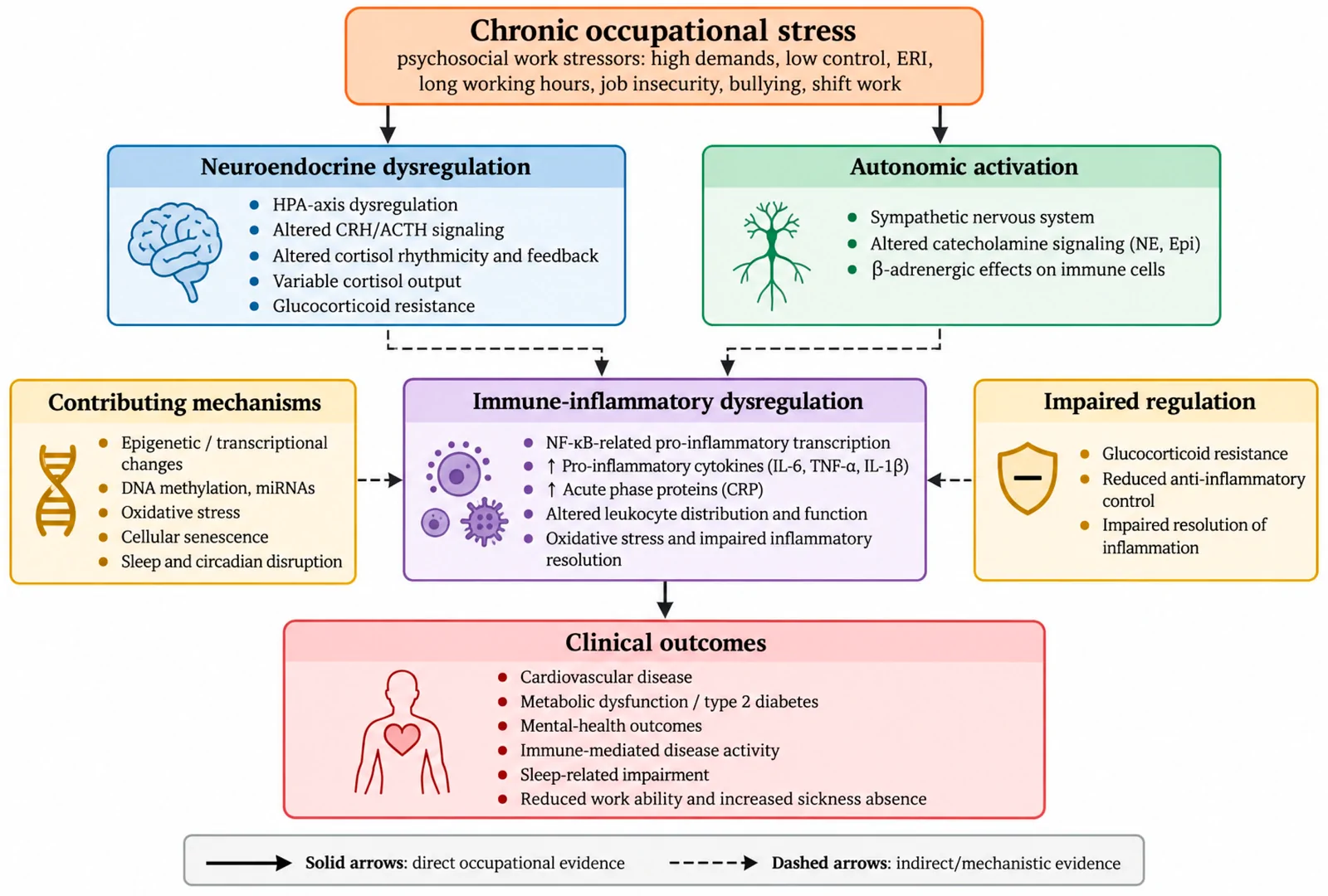
Pathophysiological pathways linking chronic occupational stress to immune–inflammatory dysregulation and disease. Graphical illustration generated with the assistance of ChatGPT (OpenAI, GPT-5.6 Sol) based on detailed author-designed scientific concepts and subsequently reviewed and approved by the authors. The arrows indicate the proposed progression from psychosocial occupational exposures toward neuroendocrine and autonomic activation, followed by impaired anti-inflammatory regulation and immune–inflammatory dysregulation. Chronic activation of these pathways may increase pro-inflammatory transcription, cytokine production, oxidative stress, and immune-cell alterations, thereby contributing to adverse clinical outcomes. The relationships shown represent biologically plausible pathways supported by evidence of varying strength and should not be interpreted as deterministic causal sequences. HPA, hypothalamic–pituitary–adrenal; CRH, corticotropin-releasing hormone; ACTH, adrenocorticotropic hormone; SNS, sympathetic nervous system; ERI, effort–reward imbalance; NE, norepinephrine; Epi, epinephrine; NF-κB, nuclear factor kappa B; IL, interleukin; TNF-α, tumor necrosis factor alpha; CRP, C-reactive protein; miRNA, microRNA. Upward (↑) arrows indicate increased biological activity or expression, respectively.

**Figure 2:**
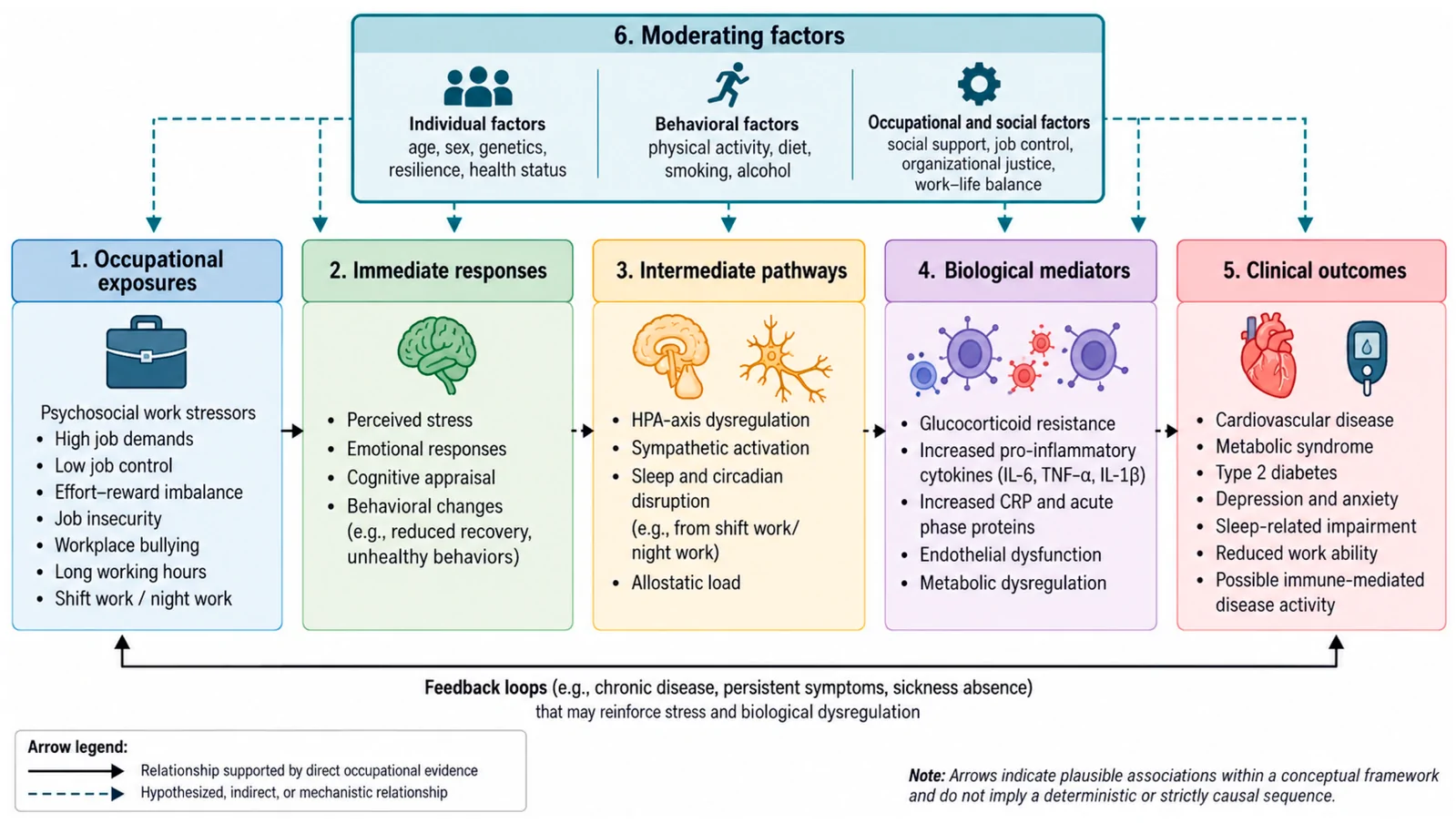
Conceptual framework linking occupational psychosocial stressors to clinical outcomes. Graphical illustration generated with the assistance of ChatGPT (OpenAI, GPT-5.6 Sol) based on detailed author-designed scientific concepts and subsequently reviewed and approved by the authors. Arrows indicate the proposed progression from workplace exposures through immediate psychological and behavioral responses, neuroendocrine and autonomic activation, and subsequent immune–inflammatory and metabolic dysregulation toward clinical outcomes. Moderating factors may strengthen or attenuate these relationships, while feedback pathways indicate that established disease, persistent symptoms, and impaired work ability may further increase psychosocial stress. The model represents potential associations rather than an obligatory causal sequence. HPA, hypothalamic–pituitary–adrenal; CRP, C-reactive protein; IL, interleukin; TNF-α, tumor necrosis factor alpha.

**Table 1:** Candidate biomarkers investigated in occupational stress research: biological functions, occupational evidence, and major limitations.

Biomarker	Main Biological Function	Evidence in Occupational Stress	Key Limitations
C-reactive protein (CRP)	Acute-phase protein and marker of systemic inflammation	Frequently investigated in occupational populations, but prospective associations are weak and heterogeneous. The prospective meta-analysis by Eguchi et al. did not identify CRP as the marker with the clearest association [5,85].	Non-specific inflammatory marker; influenced by infection, adiposity, smoking, metabolic disease, medication use, age, and other inflammatory conditions [5]. No validated occupational-stress diagnostic threshold.
Interleukin-6 (IL-6)	Pro-inflammatory cytokine involved in immune and metabolic regulation and hepatic acute-phase signaling	IL-6 showed the clearest marker-specific prospective association in the occupational meta-analysis, although the overall association was small and all included studies had serious risk of bias [5,85]. Occupational associations have also been reported in the PROspective Quebec Study and Whitehall II [5,86].	Non-specific; influenced by obesity, infection, metabolic abnormalities and other inflammatory conditions [5]. No validated occupational-stress threshold or diagnostic role.
Tumor necrosis factor-α (TNF-α)	Pro-inflammatory cytokine involved in immune-cell activation and inflammatory signaling	Occupational evidence is limited and inconsistent. TNF-α has been evaluated in occupational stress reviews, night-work studies, and law-enforcement populations, but findings are not uniform [2,47,51,85].	Not specific to occupational stress and influenced by multiple inflammatory, infectious, and metabolic conditions. No validated diagnostic role.
Interleukin-1β (IL-1β)	Major mediator of innate inflammatory responses	Direct occupational evidence is limited. Occupational studies have reported IL-1β alterations in selected worker populations, whereas stronger mechanistic evidence derives from experimental psychological-stress studies [2,21,22,51].	Short circulating half-life, methodological variability, and lack of occupational specificity. No validated occupational diagnostic application.
sICAM-1/sVCAM-1	Soluble adhesion molecules reflecting endothelial activation	Limited occupational evidence. In healthy male workers, higher work stress was associated with higher sVCAM-1 and marginally higher sICAM-1, but the study was cross-sectional and requires replication [NEW REF].	These are markers of endothelial activation, not markers specific to occupational stress. They are influenced by cardiovascular and metabolic factors and have no validated occupational diagnostic role.
Cortisol, salivary/hair	Marker of HPA-axis activity; salivary cortisol reflects short-term and diurnal activity, whereas hair cortisol may reflect longer-term cumulative exposure	Occupational findings are heterogeneous. Salivary cortisol associations vary according to stress model, work schedule, sampling time, and study population [87,88,89,90,91,92]. Hair cortisol findings are also inconsistent; only some longitudinal occupational studies reported increases, while pooled chronic-stress evidence remains heterogeneous [93,94,95].	High intra- and inter-individual variability; influenced by circadian timing, sleep, shift schedule, physical activity, medication, sampling protocol, and biological matrix [87,88,92,93,94,95]. No validated occupational cut-off.

## Data Availability

The data used to support the findings of this study are available from the corresponding author upon reasonable request.
